# NELL1, whose high expression correlates with negative outcomes, has different methylation patterns in alveolar and embryonal rhabdomyosarcoma

**DOI:** 10.18632/oncotarget.16526

**Published:** 2017-03-23

**Authors:** Lucia Tombolan, Elena Poli, Paolo Martini, Angelica Zin, Chiara Romualdi, Gianni Bisogno, Gerolamo Lanfranchi

**Affiliations:** ^1^ Department of Biology, University of Padova, Padova, Italy; ^2^ Centro di Ricerca Interdipartimentale per le Biotecnologie Innovative, University of Padova, Padova, Italy; ^3^ Institute of Pediatric Research, Padova, Italy; ^4^ Department of Women's and Children's Health, Oncology Hematology Division, University of Padova, Padova, Italy

**Keywords:** DNA methylation, RRBS, sequencing, rhabdomyosarcoma, NELL1

## Abstract

Rhabdomyosarcoma (RMS), which represents the most frequent soft tissue sarcoma in pediatric populations, is classified into two major subtypes: embryonal RMS (ERMS) and alveolar RMS (ARMS). ARMS subtype, which shows greater aggressiveness and proneness to metastasis with respect to ERMS, are characterized, in about 75% of cases, by specific chromosomal translocations that involve PAX and FOXO1 genes. Many findings have demonstrated that *PAX/FOXO1*-positive ARMS have a worse prognosis than *PAX/FOXO1*-negative ones and that distinct molecular features characterize RMS with different gene fusion statuses. DNA methylation, which presently represents a challenging research area, is involved in the modulation of gene expression.

We performed a genome-wide DNA methylation analysis using reduced-representation bisulfite sequencing (RRBS) in RMS samples and we found that fusion-positive alveolar and embryonal subgroups have different DNA methylation signatures and that ARMS fusion-positive subtypes are characterized by overall hypomethylation levels. While *NELL1* was found to be hypomethylated and transcriptionally enhanced in RMS alveolar subtypes, high *NELL1* expression levels, which proved to be correlated with negative RMS prognostic factors such as fusion status and histology (*P* < 0.0001), were found to discriminate between RMS patients with different outcomes (*P* < 0.05).

In conclusion, our results demonstrated that different DNA methylation patterns distinguish between different RMS subgroups and they suggest that epigenetic signatures could be useful for risk stratification of patients.

## INTRODUCTION

Rhabdomyosarcomas (RMS), the most common soft tissue sarcoma in pediatric populations, has been classified into two major histological subtypes: alveolar RMS (ARMS), which has a significantly worse prognosis [1], and embryonal RMS (ERMS). The cellular and molecular mechanisms underlying the greater aggressiveness and metastatic potential of ARMS with respect to ERMS are still far from being completely understood. Cytogenetic and molecular analyses have demonstrated that ARMS frequently arise from the reciprocal chromosomal translocation that involves PAX3/PAX7 genes and the transcription factor FOXO1. In addition, according to some findings, *PAX3/FOXO1*-positive ARMS have a worse prognosis than *PAX3/FOXO1*-negative ones [2, 3]. Using genome-wide approaches, some research groups including our own have demonstrated that ARMS fusion-positive patients have different molecular features with respect to RMS fusion-negative ones [4–7]. Moreover, a comprehensive genomic analysis carried out by one study examining 147 RMS tumor/normal pairs of patient specimens reported a landscape of genetic alterations in which distinct PAX3/FOXO1 -positive and -negative RMS could be distinguished [8]. Several genome-wide studies have also provided a mapping of DNA methylation in RMS [9–12]. Altogether, these studies have demonstrated that epigenetic changes such as DNA methylation are peculiar to different tumor subgroups and could be utilized to improve the accuracy of RMS diagnosis.

DNA methylation is frequently described as an epigenetic DNA modification at a promoter level that leads to reduced expression or even silencing of resident genes. Now, thanks to improved DNA methylation mapping, it has become feasible to evaluate DNA methylation in different genomic contexts other than gene promoters, such as transcriptional start sites with or without CpG islands, gene bodies, regulatory elements and repeat sequences. The picture that is emerging shows that the function of DNA methylation varies depending on the genomic context and that the relationship between DNA methylation and transcription is more nuanced than was initially proposed [13]. Alterations in DNA methylation are now known to cooperate with genetic events and to be involved in human carcinogenesis [14]. Genomic hypomethylation has generally been observed during cell transformation, resulting in an overall decrease in total genomic 5-methyl Cytosine (5mC) residues in cancer cells. Hypermethylation of CpG islands is, instead, a frequent finding that is often associated with silencing of tumor suppressor genes and downstream signaling pathways.

Carrying out a comprehensive analysis of epigenetic changes that characterize RMS subgroups, we applied, for the first time, reduced representation bisulfite sequencing (RRBS), which represents the most comprehensive sequencing method given its resolution power at a single-base level, to RMS tumors, and found that alveolar and embryonal RMS subgroups show different DNA methylation signatures and that the fusion-positive ARMS subgroup is characterized by hypomethylation. We identified *NELL1* (Neural epidermal growth factor-like 1) as a gene that is characterized by hypomethylation and transcriptional enhancing in the RMS fusion-positive alveolar subtype. Statistical analysis uncovered a correlation between *NELL1* expression and established risk factors for RMS, such as alveolar histology and positive fusion status; patients with those characteristics have, in fact, high levels of *NELL1* just as do patients with advanced tumor stages. In addition, we found that *NELL1* expression distinguishes RMS patients with different outcomes. Study results identified and defined methylation signatures that are characteristic of different RMS subtypes, something that could be useful for risk stratification of patients.

## RESULTS

### DNA methylation patterns are different in alveolar and embryonal rhabdomyosarcoma

To examine DNA methylation patterns of alveolar and embryonal RMS, we performed Illumina sequencing of 15 RMS samples using the RRBS approach, a bisulfite-based method that enriches CG-rich parts of the genome, thereby capturing the majority of promoters and other relevant regulative regions and reducing the sequencing effort required for methylation analysis. We generated an average of 65 (Mb) clean sequencing reads for each RMS sample that was mapped to the reference genome obtaining a similar distribution of reads in all the samples. This indicates that the methodology produced a highly efficient enrichment of CpG di-nucleotides and relatively unbiased RRBS library construction (Supplementary File 1A). The number of cytosine residues of promoters and CpG islands (CGI) covered by reads reached a rate of about 70–90% for each sample analyzed (Supplementary File 1B). We generated heatmaps for each RMS sample providing information about methylation distribution, CpG density distribution, and the relationship between methylation and density (Supplementary File 1C).

**Figure 1:**
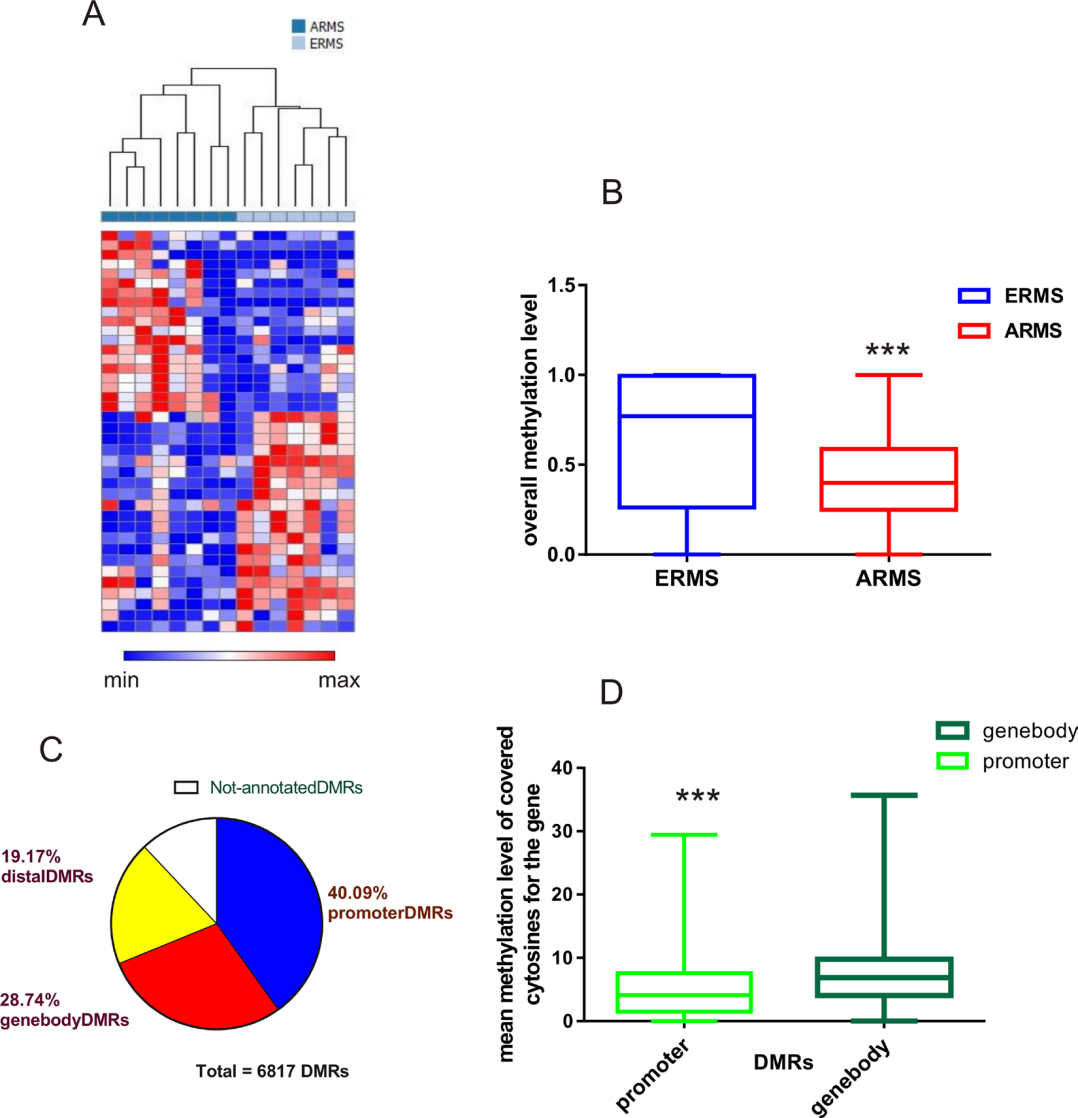
DNA methylation patterns distinguish between fusion-positive alveolar and embryonal RMS (**A**) The heatmap shows the methylation profiles of 15 RMS tumors based on unsupervised clustering. Pearson's correlation and complete linkage were applied to the top 20% of DMRs with greatest variation (based on methylation difference between two groups of samples) in the clustering analysis. (**B**) Comparison of overall methylation levels of DMRs between ARMS and ERMS. ERMS showed a significantly high rate of methylation with respect to ARMS (Mann–Whitney test ***p < 0.001). (**C**) Mapping DMRs to the genome. The majority of DMRs were localized in promoter regions, defined as regions located from -2Kb+ to 1kb to the transcription start site. DMRs associated to gene bodies and distal DMRs were also identified. (**D**) Different methylation levels of cytosine residues that were covered in different genomic regions. DMRs localized inside gene-bodies had markedly higher mean methylation levels than those located on promoters (Mann–Whitney test ***P < 0.001).

Differentially methylated regions (DMRs) were identified in ERMS and ARMS using BSmooth, a comprehensive analysis tool for whole genome datasets obtained with bisulfite sequencing [15]. We found a total of 6,817 DMRs that had different methylation values in the two groups of samples (Supplementary File 2). Methylation information can be associated to each DMR when the region is covered by at least 5 CpGs. We first selected the top 20% of DMRs whose DNA methylation level varied the most across the two groups of samples (based on the average methylation difference between the two sample groups) and then performed an unsupervised hierarchical clustering. This analysis divided the rhabdomyosarcoma samples into two groups: alveolar or embryonal (Figure 1A).

We next analyzed the overall DMRs methylation levels in the alveolar and embryonal RMS and found significantly lower levels of methylation in alveolar fusion-positive cases (*P <* 0.0001, Figure 1B). We then mapped all DMRs to the human genome (GRch37). Using the RefSeq transcript annotation (RefSEq 65), we found that about 40% of DMRs localized in promoter regions (defined as regions located from -2Kb+ to 1kb to the transcription start site, TSS), while the other DMRs mapped in intragenic regions (gene-body 28.74%) or in CpG regions distal to known coding sequences (distal DMRs, 19.17%) (Figure 1C). In addition, we observed different methylated cytosine levels in diverse genomic regions. In particular, we found that DMRs localized inside the gene bodies had markedly higher mean methylation levels than those in promoters (Figure 1D).

### Correlation of methylation and gene expression in alveolar and embryonal rhabdomyosarcoma

We focused on the genes associated to differentially methylated regions (DMRs) to gain further knowledge about the functional consequences of DNA methylation alterations in rhabdomyosarcoma. We made use of the gene expression dataset produced by Davicioni [4], the largest publicly available gene expression dataset from RMS samples. Analyzing 139 RMS samples, those investigators identified a set of differentially expressed genes in fusion-positive ARMS and RMS negative subtypes for all translocations (535 genes). Moreover, using ERMS cell line transfected with the PAX3/FOXO1 chimeric gene, they identified a signature (80 genes) that was specific for this translocation. When we compared the lists of RMS transcripts with our genomic DMR dataset to identify genes whose expression could be potentially regulated by methylation, we found that 180 genes were both differentially expressed and methylated in the ARMS and ERMS. Among them, we identified a negative correlation between methylation and RNA expression in about 50–60% of genes.

**Figure 2:**
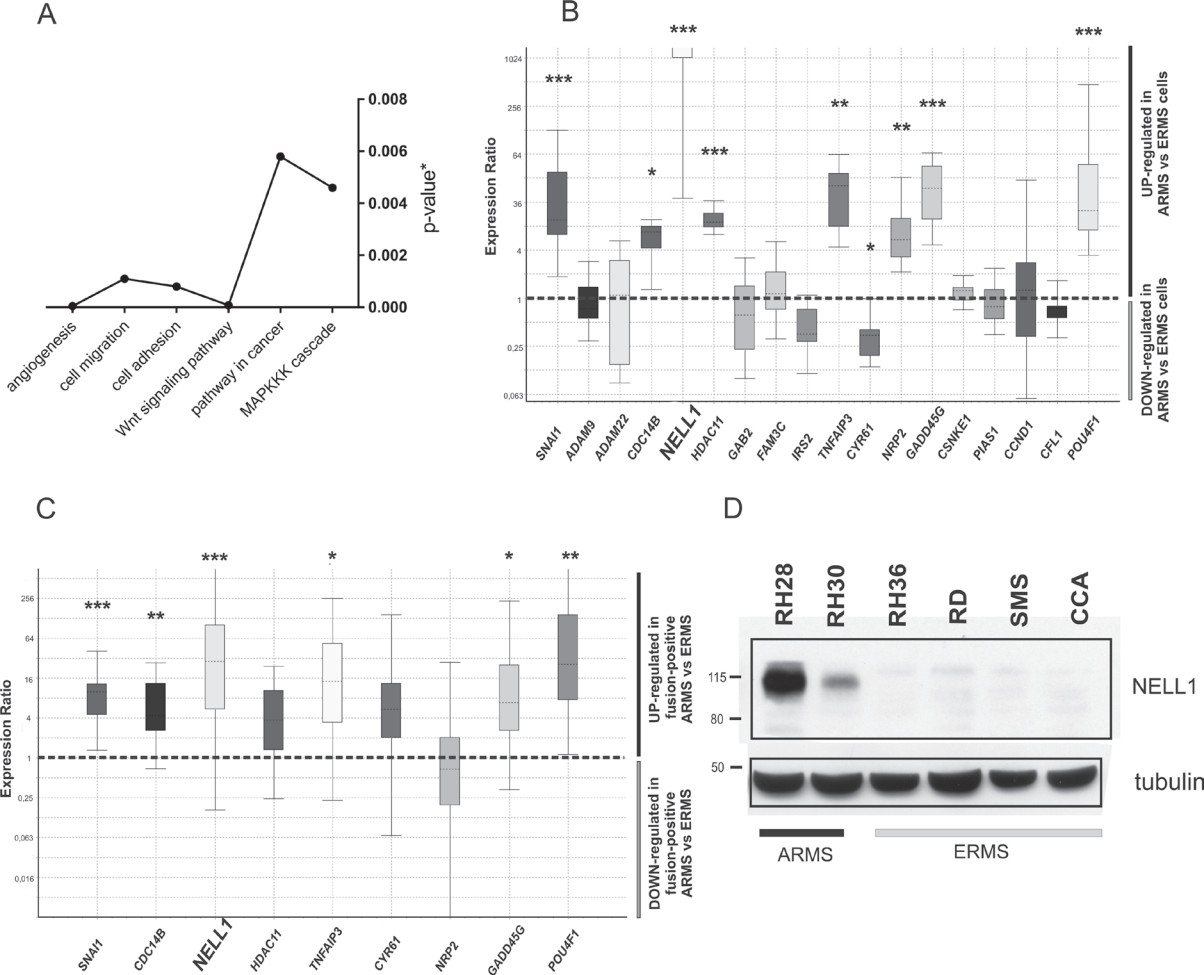
Gene expression level of methylation-related genes (**A**) Significantly represented functional classes obtained from the annotation of genes associated to the DMRs (modified Fisher exact *p-value* < 0.05). (**B**) Box plot of the relative expression levels of selected genes in ARMS cell lines compared to ERMS cell lines. (**C**) Box plot of the relative expression levels of selected genes in fusion-positive ARMS tumor samples compared to ERMS tumor samples. The samples are the same analyzed by Reduced representation bisulfite sequencing (RRBS). (**D**) NELL1 protein content was measured in whole-cell lysate of RMS cell lines and analyzed using Western blot. Glyceraldehyde-3-phosphate dehydrogenase (GAPDH) was used as housekeeping gene for qRT-PCR data normalization, and γ-tubulin as normalizer in western blot analysis. In the box plot, *P-value* were calculated by REST software *P < 0.05; **P < 0.01; ***P < 0.001.

Gene set analysis of genes that are both differentially expressed and methylated demonstrated an enrichment of Gene Ontology (GO) terms such as cell migration, cell adhesion and angiogenesis. We also found an enrichment of pathways commonly altered in cancer such as the MAPK signaling cascade and the Wnt signaling pathway (*P <* 0.001) (Figure 2A). Basing our choice on methylation and expression values and on literature data, we selected target genes belonging to enriched functional GO categories for data validation. We first assessed the expression levels of 18 selected genes in 6 RMS cell lines (4 ERMS and 2 ARMS) by qRT-PCR. Nine of them, namely *SNAI1*, *CDC14B*, *NELL1*, *HDAC11*, *TNFAIP3*, *CYR61*, *NRP2*, *GADD45G* and *POU4F1* (Figure 2B), showed significantly different expression levels in ARMS and ERMS cells. We then evaluated the expression level of those 9 genes in RMS tumor biopsies and found that *SNAI1*, *CDC14B*, *NELL1*, *GADD45G*, *TNFAIP3* and *POU4F1* were significantly different in the ARMS fusion-positive and ERMS samples (Figure 2C). *NELL1*, which emerged as the most upregulated transcript in the ARMS with respect to the ERMS cell lines, had a parallel increase in the corresponding protein (Figure 2D).

### NELL1 expression is regulated by DNA methylation in RMS cells

**Figure 3:**
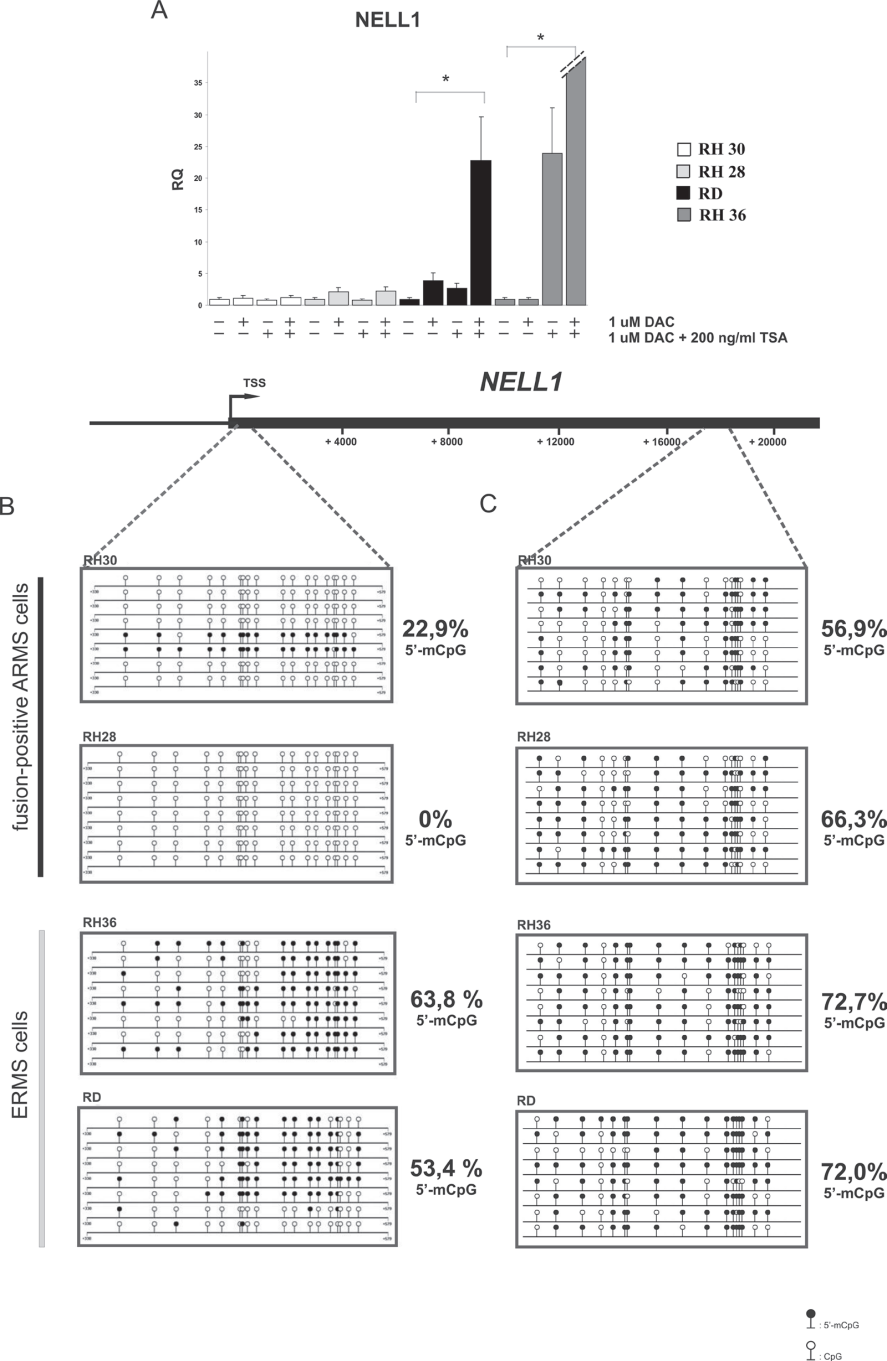
*NELL1* expression is regulated by DNA methylation (**A**) The expression of *NELL1* was rescued after treatment with 5-Aza-dC (72 h treatment) and/or with TSA (16 h treatment). The expression level was evaluated using qRT-PCR in 4 RMS cell lines (2 ARMS cell lines and 2 ERMS cell lines). DMSO-treated cells were used as control sample, while GAPDH housekeeping gene was used as normalizer. A 95% confidence interval (CI) (*p < 0.05) was calculated. Sanger bisulfite sequencing of *NELL1* promoter region (**B**) and gene-body region (**C**) revealed hypermethylation in ERMS cell lines with respect to ARMS cells. The regions investigated were identified by RRBS. 5-aza-dC: 5-aza-2′-deoxycytidine, TSA, trichostatin A, RQ, relative quantification. Circles: cytosine within CpG dinucleotides; black circles: methylated cytosine; white circles: unmethylated cytosine.

To understand whether a correlation exists between DNA methylation and gene expression we performed an *in vitro* test with known epigenetic drugs in RMS cell lines (2 alveolar RMS and 2 embryonal RMS). DNA methyltransferase inhibitor 5-Aza-2′-deoxycytidine (5-Aza-dC) and deacetylase inhibitor Trichostatin A (TSA) were used separately or in combination. We optimized the test using increasing doses of drugs reaching the best compromise between cytotoxicity and efficacy (data not shown). We analyzed the expression levels of the candidate genes described above (*GADD45G*, *POU4F1*, *NELL1 SNAI1, TNFAIP3, CDC14B)* using qRT-PCR 72 hours after treatment with 5-Aza-dC and 16 hours after treatment with TSA. We observed a restoration of expression only for the *NELL1* gene in treated ERMS cells with respect to treated control cells; no changes were observed in the alveolar cells (*P <* 0.05) (Figure 3A). Moreover, the combined use of 5Aza-dC and TSA synergistically increased mRNA expression of *NELL1* in ERMS cell lines; once again no change was observed in ARMS cell lines (Figure 3A). In order to validate the methylation pattern of *NELL1* a bisulfite Sanger sequencing was performed in the same RMS cell lines analyzed previously. Based on sequencing data, we designed two bisulfite sequencing assays: one inside the CpG island spanning the gene body and one in the CpG island that overlaps the *NELL1* promoter (Figure 3B, 3C). The specific PCR products were subcloned into bacterial vectors and used for sequencing. The results showed that the methylation levels were higher in ERMS cell lines than in ARMS cells for both those regions, but the elevation was more marked for the promoter one (Figure 3B, 3C). Taken together, these results suggest that *NELL1* expression could be regulated by epigenetic modifications in RMS cells and that different epigenetic drugs contribute synergistically to recovering gene expression.

### NELL1 involvement in migration and invasion of RMS tumor cells

**Figure 4:**
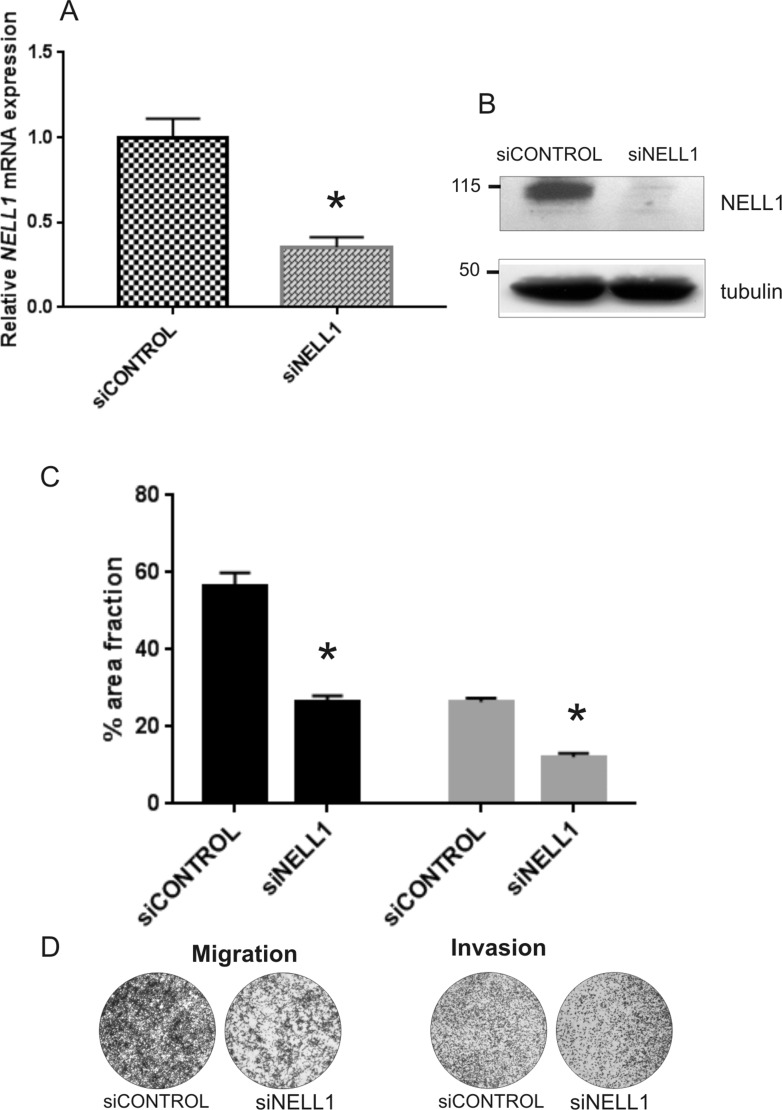
*NELL1* involvement in migration of tumor cells *NELL1* was transiently silenced using siRNA (si*NELL1*) in RH30 cells. A non-targeting siRNA was used as control (siCONTROL). The gene knock-down was evaluated using qRT-PCR (**A**) and western blot (**B**). Transwell assay on transfected and control cells was performed to evaluate the cell movement (C,D). Cell migration and invasion of *NELL1* silenced cells are shown relative to control values (siCONTROL) (**C**). An example of transwell assay is reported (**D**). Two independent experiments were performed in triplicate and mean results are shown. *P < 0.05; (The error bar represent a 95% confidence interval, IC).

To understand the role of *NELL1* in RMS, RH30, which is an aggressive ARMS fusion-positive cell line, was treated with *NELL1* siRNA. Silencing was measured 48 h post-transfection at both transcript and protein levels (Figure 4A and 4B), showing that it was really effective. Cell cycle progression and apoptosis of the transfected cells was assayed by flow cytometry but no significant changes were observed (data not shown). Conversely, when migration was evaluated by transwell assay, we noted a markedly decrease of the motility of *NELL1* silenced cells (si*NELL1*) with respect to controls (siCONTROL) (Figure 4C, 4D). In the same way, also the movement ability of the silenced RMS cells through a matrigel layer was found significantly lower respect to control cells (Figure 4C, 4D). Therefore, it seems that in RMS tumor cells *NELL1* is not affecting cell proliferation and apoptosis pathways but instead has a role in the invasive phenotype.

### High NELL1 level correlates with RMS prognostic factors

**Figure 5:**
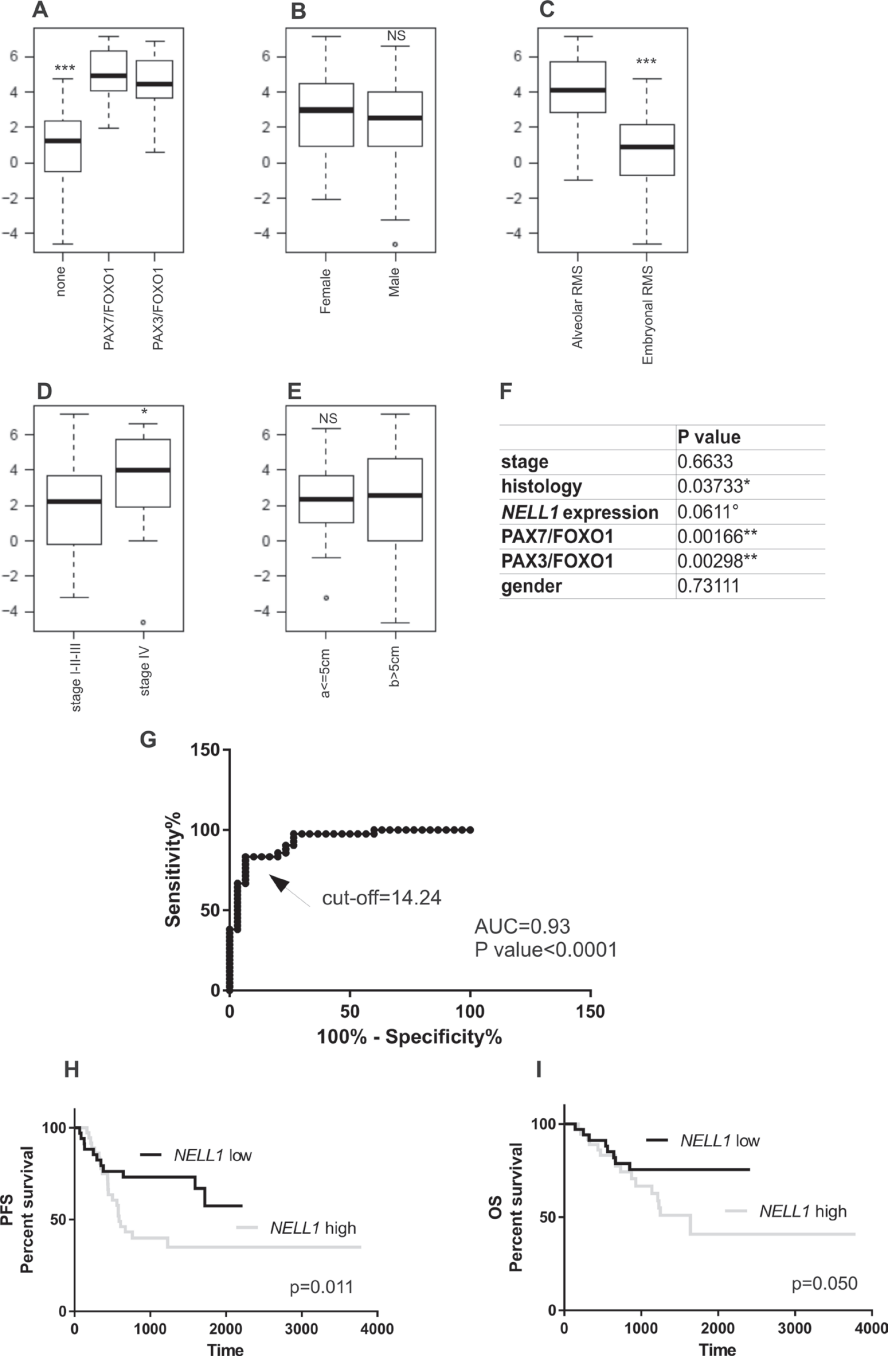
*NELL1* expression correlates with RMS clinical prognostic factors *NELL1* mRNA levels assessed by qRT-PCR showed a significant correlation with fusion status (**A**), histology (**C**) and advanced stage of disease (stage IV) (**D**), while no correlation was observed with tumor size and gender (**B**, **E**). In a multivariate analysis (Cox regression model) *NELL1* expression show a trend of correlation with known RMS risk factors (**F**). (**G**) Receiver operating characteristic curve (ROC) analysis showed the sensitivity and specificity of *NELL1* mRNA concentration as a parameter to classify RMS patients (*n* = 75) on the basis of the fusion gene status (RMS-fusion positive vs RMS-fusion negative; Cut-off RQ = 14.24). Kaplan– Meier and log-rank analysis for progression-free (PFS) (**H**) and overall survival (OS) (**I**) of RMS patients (*n* = 75) based on specific quantitative *NELL1* mRNA cutoff value (RQ = 14.24) identify two subgroups of patients with significantly different outcomes (PFS, *P* = 0.011; OS, *P* = 0.050). All statistical analyses were performed with R software and Prism6. *P < 0.05; **P < 0.01; ***P < 0.001. Abbreviations: RQ, = relative quantification; AUC = area under the curve.

The expression of *NELL1* was then assessed in an expanded cohort of RMS tumor specimens (Table 1) by qRT-PCR. To investigate if there is any correlation between *NELL1* expression and RMS patients’ outcome, we performed an association analysis with known RMS prognostic factors, such as histology, presence of translocations, tumor size and clinical stage. Data analysis of RMS patients showed that PAX/FOXO1 tumors show high levels of *NELL1* with respect to all other fusion-negative RMS (*P <* 0.001 Figure 5A), and the alveolar tumors displayed higher levels than the embryonal ones (*P <* 0.001 Figure 5B). We also observed a significantly different level of *NELL1* in patients with advanced stage disease (stage IV) with respect to counterparts with localized disease (*P <* 0.05 Figure 5D). No correlation with gender or tumor size (size > 5 cm or < 5 cm Figure 5C, 5E) was found. Multivariate analysis confirmed these data, in particular when *NELL1* was added to the list of known RMS risk factors (histology, stage, fusion status). Indeed, as expected, in our cohort of 75 patients, the histology (ARMS) and the presence of gene fusion (PAX3/7-FOXO1-positive) were significantly associated with lower survival. At the same time, high *NELL1* expression levels identifies patients with higher risk of failure, while age and tumor size at diagnosis did not appear to be significantly associated with disease outcome (Figure 5F).

**Table 1:** Main clinical characteristics of RMS patients

Variable	ARMSt (*n* = 32)	ARMSnot (*n* = 11)	ERMS (*n* = 32)
**Fusion status**			
PAX3/FOXO1	26	0	0
PAX7/FOXO1	6	0	0
**Age, years**			
< = 10	21	9	22
> 10	11	2	10
**Sex**			
Male	14	5	22
Female	18	6	10
**Site of desease**			
Parameningeal	5	3	14
Orbit	0	1	5
Head and neck	0	2	2
Extremity	18	2	3
Genitourinary	2	0	3
Bladder-prostate	0	0	4
Other sites	5	3	1
Unknown	2	0	0
**Size, cm**			
< = 5	7	3	13
> 5	19	8	19
Not evaluable	6	0	0
**IRS group**			
I	0	0	1
II	0	1	2
III	14	8	24
IV	18	2	5

Abbreviations: ARMSt= PAX3/7-FOXO1-positive alveolar rhabdomyosarcoma; ARMS not= translocation-negative alveolar rhabdomyosarcoma; ERMS= embryonal rhabdomyosarcoma; IRS= intergroup rhabdomyosarcoma study.

ROC curve analysis taking into account patients’ histology (alveolar or embryonal) and the fusion gene status (*PAX3/7-FOXO1* expression) was also carried out. The ROC curve made it possible to identify a cutoff value of *NELL1* mRNA expression. We thus demonstrated that a *NELL1* cutoff level was capable of accurately distinguishing (sensitivity = 83.33, CI = 68.64–93.03; specificity 93.33, CI = 77.93-99.18) between PAX3/7-FOXO1-positive RMS and fusion gene-negative tumors (Figure 5G, AUC = 0.93, CI 95%, *P <* 0.0001). The prognostic value of *NELL1* quantification was substantiated by Kaplan–Meier analysis for progression free survival (PFS) and overall survival (OS). RMS patients with low levels of *NELL1* transcript (relative quantification RQ < 14.24) had a significantly reduced risk of failure with respect to those showing high *NELL1* expression (Figure 5H, 5I, log-rank test, PFS *p* = 0.011, OS *p* = 0.05).

## DISCUSSION

The current study investigated the methylation signatures of genomic DNA in alveolar and embryonal rhabdomyosarcoma using a next-generation sequencing approach, which is able to achieve fine mapping of DNA methylation patterns with single-base coverage. Our results demonstrated that there are epigenetic differences between fusion-positive alveolar and embryonal rhabdomyosarcomas suggesting that DNA methylation could be an additional factor contributing to define the molecular profile of different RMS tumor subgroups. The study also investigated how DNA methylation modulates gene expression, and it identified *NELL1* (Neural epidermal growth factor-like 1) as a novel gene whose expression is correlated with known RMS prognostic factors and disease outcomes.

Other research groups as well as our own have shown that *PAX/FOXO1* positive RMS and *PAX/FOXO1* negative RMS have different gene expression profiles [4, 5]. Some of these expression alterations are due to the PAX3/PAX7–FOXO1 fusion protein acting as a transcription factor modulating the expression of downstream targets. Other gene expression differences between fusion-positive and fusion-negative RMS could be linked to additional genetic alterations, such as point mutations, gene amplification [8] or epigenetic factors including miRNAs and DNA methylation regulation [16, 9]. Illumina arrays also demonstrated that DNA methylation profiles distinguish between fusion-positive and fusion-negative samples [10, 11]. Based on the next-generation approach, our results confirmed that RMS subtypes have different methylation patterns. We also noted different overall methylation levels in ARMS fusion-positive and ERMS samples. Although the mechanisms underlying the alterations of DNA methylation are unclear, increased levels of DNA methyltransferase have been associated with different types of cancer [17, 18]. An increase in DNA methyltransferase expression has also been observed in rhabdomyosarcoma samples with respect to that in normal skeletal muscle ones. Furthermore, differential activity of DNA methyltransferase was found in the two major subgroups of RMS and the expression was higher in the embryonal subtype [19]. The different overall methylation levels that we observed in ARMS and ERMS samples could thus be linked to diverse DNA methyltransferase activities and to cross-talk between PAX3/FOXO1 signaling and DNA methylation. DNA methylation has recently been shown to have various types of relationships with transcription in specific genomic contexts [20]. The great majority of studies have focused on methylation of promoter regions adjacent to the transcription start site (TSS) where transcription initiation is generally blocked. Genome-wide approaches have revealed that methylation in gene bodies does not block transcription elongation [21] but may instead play a role in regulating RNA splicing. One study recently reported that CpG islands located in gene bodies or in intragenic regions can be preferential sites for *de novo* methylation in cancer [22]. Interestingly, when we mapped the DMRs to the genome, we observed that 40% overlap with promoter regions (defined as 2 kb upstream and 1kb downstream of the RefSeq transcription star site) while the remaining 28.74% and 19.17% mapped, respectively, within the gene body or in intergenic regions. In addition, we found that DMRs localized within gene bodies had markedly higher mean methylation levels with respect to promoter DMRs, suggesting that different genomic regions show distinct methylation patterns reflecting diverse functional roles.

We matched the DMRs identified with RMS public gene expression data to study how epigenetic modifications affect gene expression in RMS subtypes [4]. Our results support the hypothesis that epigenetic regulation is an important mechanism in tumorigenesis modulating mRNA expression, thereby contributing to differential gene expression in ARMS fusion-positive and ERMS. Interestingly, we noted an overall inverse correlation between methylation and RNA expression in about 50% of genes; this would suggest that the relationship between DNA methylation and transcription does not always follow the classical paradigm of inverse correlation. Our results showed that DNA methylation regulates *NELL1* expression in rhabdomyosarcoma. *NELL1* gene encodes a protein kinase C binding protein that contains six EGF-like domains and belongs to a new class of cell signaling molecules controlling cell growth and differentiation [23, 24]. *NELL1* emerged as a target gene regulated by PAX3/FOXO1 fusion protein from a gene expression analysis [4]. Findings from the current study demonstrated that *NELL1* is not only differentially expressed in fusion-positive ARMS and ERMS, but that its expression is modulated by DNA methylation.

Tissue-specific gene expression levels are available in public databases such as the Genotype-Tissue Expression project (GTEx). It was found that *NELL1* is a gene expressed at high levels in the brain, while other tissues, including muscle, show low expression. Our results confirmed that there is a low expression of *NELL1* in fetal skeletal muscle (FSM), which we used as a calibrator in qRT-PCR experiments, and uncovered basal levels of *NELL1* in ERMS samples. Higher *NELL1* expressions were found in fusion-positive ARMS. Moreover, our preliminary functional data on RMS cells highlighted the potential role of *NELL1* as an oncogene in RMS. In fact, we observed that *NELL1*-silenced cells decrease significantly their migratory capability.

Our findings support those outlined by Rapa et al. who demonstrated an overexpression of *NELL1* in ARMS cells with respect to ERMS cell lines. They also demonstrated that an overexpression of *NELL1* significantly increased invasion in primary myoblasts suggesting that *NELL1* could act as an oncogene in RMS [25]. Conversely, a high frequency of *NELL1* promoter hypermethylation was found in several solid tumors suggesting that *NELL1* inactivation could be involved in cancer progression [26–28]. Taken together, these finding suggest that *NELL1* acts in different manners in diverse cancer types and that further investigation is warranted to clarify its function.

We have also provided the first evidence indicating that *NELL1* mRNA levels, measured using quantitative RT–PCR, could be useful in stratifying these patients as it is significantly higher in RMS tumors characterized by adverse clinicopathological parameters, such as an unfavorable PAX3/7-FOXO1-postive histology and advanced disease stage, and they are correlated with patients’ increased risk of relapse and lower survival. In this context, high *NELL1* mRNA levels identifies patients with higher risk of failure and poor outcome and accurately distinguishes between PAX3/7-FOXO1-positive ARMS and less aggressive PAX3/7-FOXO1-negative tumors. Although *NELL1* mRNA was not found to be a strong prognostic marker in a multivariate analysis examining RMS clinical risk factors, its expression may be predictive of an unfavorable phenotype in primary RMS.

Finally, our study suggest that epigenetic regulation of specific genes could represent a novel therapeutic target that might enhance the efficacy of RMS treatment. At now several DNA methyltransferases and histone deacetylases inhibitors are FDA-approved anti-cancer drugs used in different solid tumors. In particular, EZH2 inhibitor is currently used in a phase 1 study in pediatric synovial sarcoma highlighting that epigenetic compounds could be used also in pediatric sarcomas.

In conclusion, the current study provides fine-mapping of DNA methylation of the two major subgroups of rhabdomyosarcoma and indicates how epigenetic patterns characterize different RMS subtypes. The results demonstrate that *NELL1* is differentially expressed in fusion-positive ARMS and in ERMS samples, and they show that this transcriptional difference partially depends on genomic DNA methylation. The study also found that high *NELL1* levels are correlated with negative RMS prognostic factors and with poor tumor outcomes. Finally, it confirmed that epigenetic regulation plays a key role in modulating gene expression in rhabdomyosarcoma, and suggests that DNA methylation signatures could be useful for risk stratification of these patients.

## MATERIALS AND METHODS

### Cell culture

RMS cell lines were maintained in Dulbecco's modified Eagle's medium containing 10% fetal calf serum, penicillin and streptomycin (100 ug/mL) (Life Technologies, Carlsbad, CA) at 37°C in 5% CO_2_ in a humidified incubator. RH30, RD cells were obtained from the American Type Culture Collection (Manassas, VA); CCA was a gift from Prof. Pier Luigi Lollini (Dept. Medicina Specialistica, Diagnostica e Sperimentale, University of Bologna, Italy) [29]. RH28 were a gift from Dr. Peter J. Houghton (St. Jude Children's Hospital, Memphis, TN) [30]. RH36 was provided by Dr. Maria Tsokos (National Cancer Institute, Bethesda, MD) [31]. RMS cell lines features are summarized in Supplementary File 3.

### Tumor samples and ethics issues

Tumor biopsies were received from the Italian Association of Pediatric Hematology and Oncology Soft Tissue Sarcoma Bank at the Department of Women's and Children's Health, University of Padova (Padova, Italy). This study was approved by the local Ethics Committee as part of a clinical trial currently carried out with the Associazione Italiana Ematologia Pediatrica (AIEOP = Italian Association of Pediatric Hematology and Oncology). Relevant clinical data of patients enrolled in the study is reported Supplementary File 4.

### Total RNA and DNA isolation

Genomic DNA was isolated from RMS cell lines and from RMS tumor biopsies using Qiamp DNA mini Kit (Qiagen) following manufacturer's instructions. RNA was isolated from RMS specimens using Trizol^®^ Reagent (Life Technologies) or with AllPrep DNA/RNA/protein kit (Qiagen) following manufacturer's instructions.

### Reduced-representation bisulfite sequencing

Reduced-representation bisulfite sequencing (RRBS) is a bisulfite-based protocol that enriches CG-rich parts of the genome, thereby reducing the amount of sequencing required while capturing the majority of promoters and other relevant genomic regions. The RRBS sequencing of our RMS samples was performed by the Beijing Genomics Institute (BGI) using an Illumina HiSeq2000 sequencer. The pipelines of experimental procedures and bioinformatics analysis are summarized in the Supplementary File 5. Cytosines observed on the forward read of each read pair were in silico replaced by thymines; and guanines observed on the reverse read of each read pair were replaced in silico by adenines [32]. The “fastq” sequencing reads were mapped to the reference by Short Oligonucleotide Analysis Package (SOAP) aligner. Every hit with a single placement with minimum numbers of mismatches and a clear strand assignment was defined as unambiguous alignment (uniquely mapped reads). Uniquely mapped reads with restriction enzyme cut sites were used for further analysis.

BSmooth software, an open source software available at https://www.rdocumentation.org/packages/bsseq/versions/1.8.2/topics/BSmooth [15], was used to identify differentially methylated regions (DMRs) from the two major subtypes, i.e., alveolar and embryonal RMS. The DMR lists and bioinformatics analysis of the sequencing performed by the BGI are available in the Supplementary Files 1, 2.

We developed a pipeline to associate the DMRs to the promoter region (promoter DMRs), to the gene body (gene-body DMRs) and to distal upstream region (distal DMRs). The classes were defined as follows: “promoter”, from −2000 to +1000 relative to transcriptional start site (TSS); “gene-body”, the complete span of the exons of the transcripts; and “distal”, from −50000 to −2000 relative to TSS. These regions were defined for all transcripts in RefSeq and were intersected with the DMRs. In the event of multiclass assignments, we assigned the DMR to one class following this priority: 1 = promoter, 2 = gene-body, 3 = distal. The cases of multiple transcripts assigned to the same DMR of interest were manually evaluated individually. The mean methylation levels of covered cytosines for each DMR/sample was used as the methylation value to perform a heatmap by GENE-E (Broad Institute). All statistical analyses were performed using Prism6 software, and the Mann-Whitney test and/or t-Student test was applied.

### Expression analysis

Gene expression data were obtained from the appropriate literature. We used differentially expressed gene datasets identified by Davicioni et al. [4] during their extensive study of gene expression profiling of 139 RMS samples using Affymetrix chips. The genes that were both differentially expressed and methylated underwent functional analysis using Gene Ontology (GO) implemented by the DAVID tool [33]. The significantly enriched biological categories were identified using the Modified Fisher Exact *p-value* < 0.05.

### Availability of data and materials

Raw data are available on the Sequence Read Archive (SRA) website using accession number SRP078221, and the data processed are presented as Supplementary Supporting Files.

### qRT-PCR for mRNA detection

qRT-PCR and statistical analysis of expression data were performed as described in ref [12].

### Trichostatin A and 5-aza-2′-deoxycytidine treatments

TSA and 5-Aza-dC treatments were performed as previously described in ref [12].

### Sodium bisulfite treatment of DNA and bisulfite sequencing

The sodium bisulfite treatment and converted DNA sequencing were as reported previously [12].

### Immunoblotting

Cells were washed twice in 1 X PBS and incubated on ice for 20 min with lysis buffer composed of 20 mM Tris–HCl (pH 7.5), 140 mM NaCl, 25 mM NaF, 25 mM glycerophosphate, 5 mM sodium orthovanadate, 5 mM, EDTA, 5 mM EGTA, 1 mM sodium PP (pH 7.0), 1 mM DTT, 1.5 mM MgCl2, 10% glycerol, 1 mM phenylmethyl–sulfonyl fluoride (PMSF), 20 ug/ml leupeptin, 20 ug/ml aprotinin and 1% Triton X-100. Cell lysates were clarified by centrifugation at 4°C at 14 000 × g for 30 min. The protein concentration was determined by the bicinchoninic acid. Cell lysates were fractionated by 10% SDS–PAGE transferred to nitrocellulose membranes and visualized using specific antibodies against NELL1 (1:2000, GeneTex) and ƴ-tubulin (1:6000).

### RNA interference

To obtain transient siRNA expression, RH30 were plate at 80% confluence and transfected using Lipofectamine2000 transfection reagent (Thermofisher Scientific) with 50 nM siRNA for *NELL1* (si*NELL1*). As control we transfected the cells with a 50 nM of non-targeting siRNA (siCONTROL). The gene knockdown was evaluated at the mRNA level using qRT-PCR and at protein levels by Western Blot after 48 hours from transfection.

### Transwell assay

To measure migration and chemoinvasion, 1,2*10^5^ RH30 cells per well were seeded in serum-free medium in the upper chambers of cell culture transwells with 8.0-μm pore size membrane, or in the upper chambers of cell culture transwell with BioCoat Matrigel (24-well format; Becton Dickinson BD Biosciences). The lower chambers were filled with 0.75 ml of complete growth medium or with serum-free Dulbecco's modified Eagle's medium as a control. After 21 hours at 37°C in a humidified incubator with a 5% CO2 atmosphere, the migrated cells on the lower side of the membrane were fixed in 2.5% glutaraldehyde, stained with 0.2% crystal violet and photographed using a stereomicroscope (NIKON SMZ1500) equipped with a CCD camera. Images were processed with CorelDraw software (Corel, Ottawa, Canada), and ImageJ software (http://rsbweb.nih.gov/ij) was used to quantify the area covered by the migrated cells. A 95% Confidence interval (CI) was calculated.

### Statistical analysis

The patients included in the study had a median follow-up time of 2.8 years after diagnosis (range 0.06–10.3 years). The association of NELL1 expression levels with specific clinical and molecular variables was analyzed either by Student's *t-test* or the Mann–Whitney *U-test* in the event the variables did not satisfy normality. Kaplan–Meier survival curves with log-rank test and Cox proportional hazard regression model were applied to evaluate the prognostic potential of NELL1 expression of RMS patients, respectively, in univariate and multivariate analysis. Receiver-operator characteristic curve (ROC) was calculated to define the best cutoff values of *NELL1* predicting events or fusion gene status in terms of sensitivity and specificity. Overall survival (OS) was calculated from the date of diagnosis to the date of death for any cause or the last follow-up; progression-free survival (PFS) was calculated from the date of diagnosis to the date of the first event (tumor progression or relapse) or the last follow-up. The analyses were performed using Prism6 and R statistical software.

## SUPPLEMENTARY MATERIALS FIGURES AND TABLES







## Abbreviations

RMS: rhabdomyosarcoma
ARMS: alveolar rhabdomyosarcoma
ERMS: embryonal rhabdo- myosarcoma
CpG: cytosine-phosphate-guanine nucleotide sequence
DMRs: differentially methylated regions
GO: gene ontology
qRT-PCR: quantitative real time polymerase chain reaction
RRBS: reduced representation bisulfite sequencing
NELL1: Neural epidermal growth factor-like 1
5-aza-dC: 5-aza-2′-deoxycytine
TSA: trichostatin A.
