# Supplementary material for: NELL1, whose high expression correlates with negative outcomes, has different methylation patterns in alveolar and embryonal rhabdomyosarcoma
Source: Oncotarget. 2017 Mar 23;8(20):33086–99. doi: 10.18632/oncotarget.16526 (PMC5464852; doi:10.18632/oncotarget.16526)
Supplement: Supplementary file 1 [file oncotarget-08-33086-s001.pdf]

NELL1, whose high expression correlates with negative outcomes, has different methylation patterns in alveolar and embryonal rhabdomyosarcoma

Supplementary Materials

Supplementary File 1: RRBS sequencing analysis

A) Result of reads alignment

Sample ID	Clean reads (M)	Mapped reads (M)	Map rate (%)	Uniquely mapped	Uniquely mapped	Enzyme cutting	Enzyme rate (%)	Bisulfite conversion
RMS 1	65.31	60.34	92.40	45.56	69.77	44.09	96.77	99.78
RMS 2	65.31	58.92	90.23	44.55	68.22	43.13	96.80	99.77
RMS 3	65.31	89.40	90.96	44.03	67.42	43.20	98.12	99.82
RMS 4	65.31	59.35	90.87	45.38	69.48	43.81	96.53	99.79
RMS 5	65.31	59.97	91.83	45.29	69.35	44.15	97.48	99.80
RMS 6	68.50	60.65	88.54	45.57	66.53	44.20	97.00	99.77
RMS 7	65.31	59.97	91.83	45.82	70.16	44.89	97.98	99.78
RMS 8	64.05	59.79	93.34	45.73	71.40	44.84	98.05	99.41
RMS 9	65.31	58.58	89.70	43.52	66.63	42.27	97.15	99.47
RMS 10	65.31	57.27	87.69	41.98	64.29	41.10	97.90	99.33
RMS 11	65.31	59.98	91.84	46.25	70.82	45.29	97.92	99.49
RMS 12	65.31	51.64	79.07	37.98	58.16	36.20	95.32	99.18
RMS 13	65.31	59.72	91.45	45.53	69.71	44.42	97.57	99.45
RMS 14	65.31	59.20	90.65	44.78	68.56	43.75	97.71	99.76
RMS 15	65.31	58.61	89.75	43.25	66.22	42.19	97.55	99.80

B) Coverage of cytosine per sample

		Promoter			CGI		
		CG	CHG	CHH	CG	CHG	CHH
Genome		3,638,243	5,193,077	13,173,586	4,179,076	3,633,771	6,769,239
Target region		1,535,708	1,654,557	3,276,635	2,129,692	2,069,239	3,810,191
RMS 1	number	1,166,468	1,208,293	2,237,289	1,608,900	1,541,531	2,714,517
	rate (%)	75.96	73.03	68.28	75.55	74.50	71.24
RMS 2	number	1,205,037	1,261,026	2,357,999	1,653,92	1,592,256	2,820,950
	rate (%)	78.47	76.22	71.96	77.66	76.95	74.04
RMS 3	number	1,143,841	1,177,860	2,157,440	1,577,656	1,512,161	2,644,878
	rate (%)	74.48	71.19	65.84	74.08	73.08	69.42
RMS 4	number	1,163,39,1	1,200,297	2,218,403	1,603,684	1,533,411	2,695,155
	rate (%)	75.76	72.54	67.70	75.30	74.11	70.74
RMS 5	number	1,209,461	1,272,206	2,358,794	1,662,463	1,612,002	2,837,066
	rate (%)	78.76	76.89	71.99	78.06	77.90	74.46
RMS 6	number	1,185,791	1,240,269	2,297,223	1,632,231	1,576,804	2,772,697
	rate (%)	77.21	74.96	70.11	76.64	76.20	72.77
RMS 7	number	1,170,499	1,218,749	2,259,193	1,607,911	1,546,622	2,721,212
	rate (%)	76.22	73.66	68.95	75.50	74.74	71.42
RMS 8	number	1,197,100	1,235,516	2,284,678	1,647,487	1,575,325	2,773,042
	rate (%)	77.95	74.67	69.73	77.36	76.13	72.78
RMS 9	number	1,179,974	1,223,603	2,245,719	1,620,345	1,560,341	2,725,868
	rate (%)	76.84	73.95	68.54	76.08	75.41	71.45
RMS 10	number	1,068,256	1,078,697	1,890,738	1,480,603	1,416,881	2,400,287
	rate (%)	69.56	65.20	57.70	69.52	68.47	63.00
RMS 11	number	1,206,919	1,273,752	2,373,528	1,652,697	1,606,141	2,835,124
	rate (%)	78.59	76.98	72.44	77.60	77.62	74.41
RMS 12	number	882,683	894,584	1,552,105	1,216,030	1,180,208	1,980,914
	rate (%)	57.48	54.07	47.37	57.10	57.04	51.99
RMS 13	number	1,196,924	1,250,537	2,312,704	1,646,981	1,589,145	2,791,320
	rate (%)	77.94	75.58	70.58	77.33	76.80	73.26
RMS 14	number	1,136,344	1,176,323	2,162,852	1,565,851	1,506,941	2,638,405
	rate (%)	73.99	71.10	66.01	73.52	72.83	69.25
RMS 15	number	1,135,871	1,176,829	2,155,989	1,564,133	1,508,636	2,632,440
	rate (%)	73.96	71.13	65.80	73.44	72.91	69.09

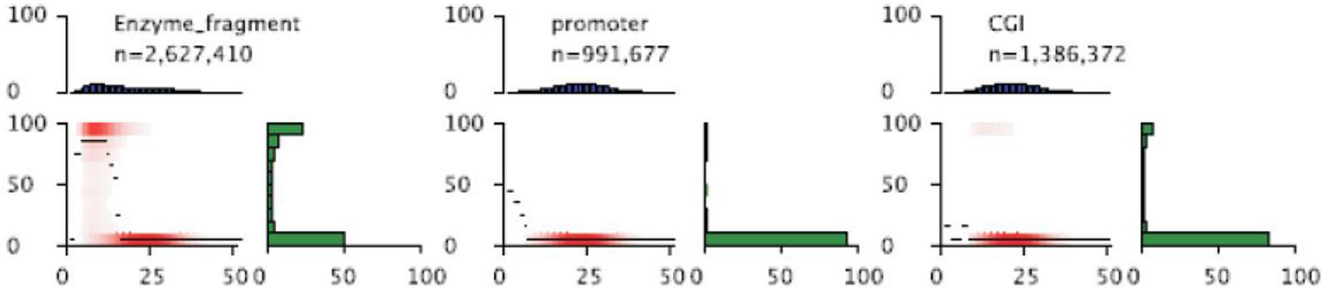

(C) Heatmaps show distinct methylation and CpG density patterns. Each panel represents a separate feature, and 'n' refers to the numbers of analyzed CpGs (per-strand depth ≥ 4) within that feature. CpG density (x-axis) is defined as numbers of CpG dinucleotides in 200 bp windows. Methylation level (y-axis) is defined as average methylation level of cytosines in CpGs. The thin black lines within each heat map denote the median methylation level of cytosines CpGs at the given local density. The red color gradient indicates abundance of CpGs that fall into bins of given methylation level and CpG densities. The blue bar charts above each heat map show the distribution of CpG densities, projected onto the x-axis of the heat maps. The green bar charts to the right of the heat maps show the distribution of methylation levels, projected onto the y-axis of the heat maps.

Supplementary File 2: List of *differentially methylated regions* (DMRs) in ERMS and ARMS. See Supplementary_File_2

Supplementary File 3: Summary of features of RMS cell lines

Cell lines	Karyotype/Gene fusion status	Hystology	Origins
RH28	t(2;13)(p25;q14);near tetraploid	Alveolar	Axillary metastasis 17-year-old male
RH30	t(2;13)(p25;q14), TP53 mutation; amplification of 12q13-15 region including CDK4	Alveolar	Bone marrow metastasis 16-year-old male
RH36	Unknown	Embryonal	Paratesticular relapse 15-year-old male
RD	51-hyperdiploid; MYC amplification; Q61H mutation of NRAS; TP53 mutation	Embryonal	Pelvic mass 7-years-old female
CCA	multiple chromosomal rearrangements; Q61L mutation of KRAS	Embryonal	Vescical mass 8 years old male
SMS-CTR	Hypertriploid	Embryonal	Pelvic mass 1-years old male

Supplementary File 4: Clinical characteristics of the RMS patients analyzed. See Supplementary_ File_4

Supplementary File 5: Pipelines of experimental procedures and bioinformatics analysis of RRBS sequencing

Pipeline of experiment

The pipeline of experiment is illustrated in the figure below. The DNA sample will have the following treatment after passing the sample quality test:

1. Genome DNA were cut by Restriction Enzyme
2. DNA-end repair, 3'-dA overhang
3. Select the 40-220 bp fragment
4. Bisulfite treatment by ZYMO EZ DNA Methylation-Gold Kit
5. PCR amplification
6. Qualified library for sequencing

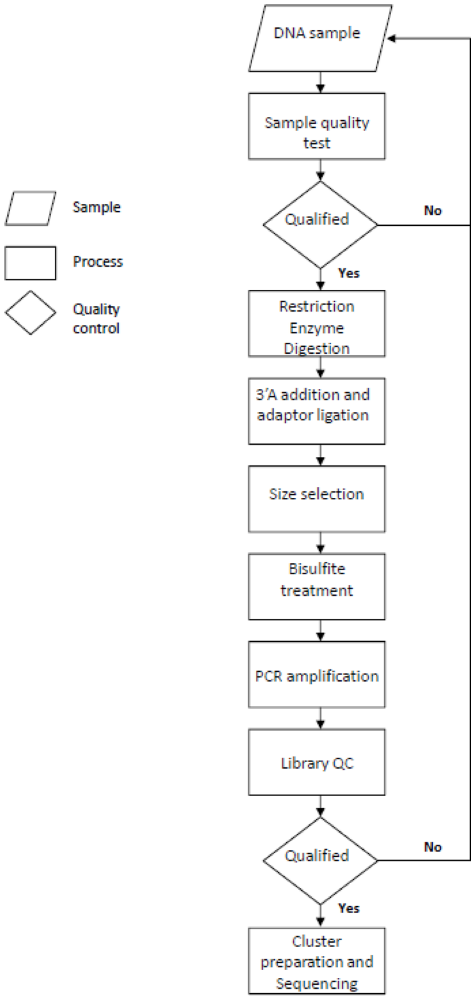

Pipeline of experiment. After the DNA sample(s) was(were) delivered, we did a sample quality test first. We used this(those) qualified DNA sample(s) to construct RRBS library, then we did a library quality test. At last, the qualified RRBS library would be used for sequencing.

Pipeline of bioinformatics analysis

Sequencing data will be mapped to reference genome. Only the uniquely mapped reads can be used for standard analysis and personalized bioinformatics analysis. The pipeline of analysis is as follows:

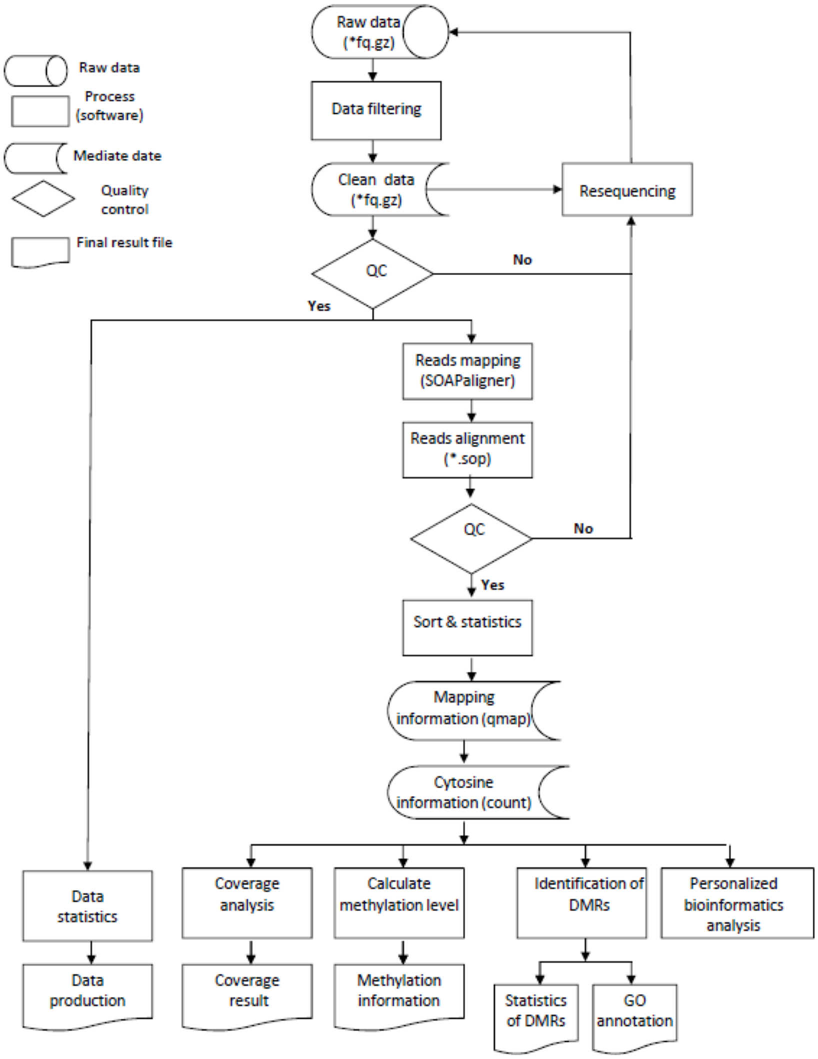

Pipeline of Bioinformatics Analysis. After sequencing data was delivered, we did data filtering first, which could remove those low-quality data, then, we mapped the clean data to reference genome if clean data was qualified. Also, we needed to make a quality test about the alignment. We used those uniquely mapped reads which have enzyme cutting site to get methylation information of cytosine if the alignment result was qualified. After that, we will do the coverage analysis and methylation analysis. Furthermore, DMR analysis if there are multiple samples.
